# BMI-specific associations between health-related behaviours and overweight – a longitudinal study among Norwegian adolescents

**DOI:** 10.1017/S1368980016002536

**Published:** 2016-09-22

**Authors:** Inger M Oellingrath, Martin V Svendsen

**Affiliations:** 1 Faculty of Health and Social Sciences, Department of Health Studies, University College of Southeast Norway, PO Box 201, 3914 Porsgrunn, Norway; 2 Department of Occupational and Environmental Medicine, Telemark Hospital, Skien, Norway

**Keywords:** Health-related behaviour, Overweight, Adolescents, BMI-specific, Longitudinal

## Abstract

**Objective:**

To investigate BMI-specific associations between health-related behaviours in early adolescence and the likelihood of overweight in mid-adolescence in a sample of Norwegian adolescents.

**Design:**

Longitudinal study of 393 adolescents recruited from schools in Telemark County, Norway. Parents reported baseline data on children’s behavioural variables and background data (at age 12–13 years). Dietary data were reported by means of a retrospective FFQ. Eating patterns were identified using principal component analysis. Height and weight were measured at baseline and 3-year follow-up. BMI-specific differences in health-related behaviours (eating patterns, physical activity and screen time) at baseline were analysed using cross-tabulation and Pearson’s *χ* ^2^ test (Fisher’s test). Associations between early health-related behaviours and the likelihood of later overweight were examined using multiple logistic regression.

**Setting:**

Primary and secondary schools, Telemark, Norway.

**Subjects:**

Children (*n* 393) in 7th grade (mean age 12·7 (sd 0·3) years), followed up in 10th grade, and parents.

**Results:**

A moderate to high intake of a varied Norwegian eating pattern combined with moderate-to-vigorous physical activity ≥1 h/d in the 7th grade were associated with a reduced likelihood of being overweight in the 10th grade, but only among already overweight adolescents (adjusted OR=0·2; 95 % CI 0·1, 1·0). Screen time of >3 h/d in the 7th grade was associated with an increased likelihood of subsequent overweight only among adolescents with an initial normal weight (adjusted OR=2·8; 95 % CI 1·1, 7·3).

**Conclusions:**

BMI-specific associations were observed between health-related behaviours in early adolescence and the likelihood of being overweight in mid-adolescence.

Overweight and obesity among children and adolescents present a significant health problem worldwide^(^ ^1^ ^,^ ^2^ ^)^. Health-related behaviours such as high intake of energy-dense and low-fibre foods, low physical activity and screen-based sedentary behaviour are believed to predispose young people to later overweight and obesity^(^ ^3^ ^–^ ^7^ ^)^. This evidence underlies worldwide public health advice and intervention programmes designed to prevent overweight and obesity development in children and adolescents.

In Norway, overweight and obesity are also excessively prevalent among adolescents and represent a major public health concern^(^ ^8^ ^)^. As in most developed countries^(^ ^9^ ^–^ ^11^ ^)^, a significant proportion of Norwegian children and adolescents fail to meet national guidelines on diet and physical activity, eating fruit and vegetables too seldomly, consuming energy-rich and sugar-rich products too frequently, and failing to engage in the recommended 1 h of moderate-to-vigorous physical activity (MVPA) daily^(^ ^12^ ^)^. According to national data, sedentary activities like television viewing and computer use have also increased markedly among Norwegian children and adolescents over the last decade^(^ ^13^ ^)^.

Both healthy and unhealthy behaviours may track from childhood to adolescence and on into adulthood^(^ ^14^ ^–^ ^18^ ^)^. Early adolescence is considered a crucial phase during which the incidence of potentially obesogenic behaviours often increases^(^ ^4^ ^,^ ^5^ ^,^ ^13^ ^,^ ^19^ ^)^. This makes the period particularly important in terms of recognising health-related predictors of subsequent overweight and implementing adequate measures to promote healthy, enduring habits. There is currently a general lack of Norwegian longitudinal studies exploring associations between health-related behaviours and overweight among adolescents. More studies, preferably incorporating objectively measured BMI, are needed^(^ ^20^ ^)^.

Previous studies have suggested that longitudinal relationships between health-related behaviours (overall diet, physical activity and screen time) and overweight/obesity in children and adolescents may be modified by initial BMI status^(^ ^7^ ^,^ ^18^ ^,^ ^21^ ^–^ ^25^ ^)^. Current knowledge indicates that overweight and normal-weight young people may respond differently, in terms of weight change, to changes in diet, physical activity and sedentary behaviour. However, relatively few BMI-stratified longitudinal studies of young people have been conducted and additional studies would be useful to add knowledge to the field. Knowledge of behaviours in early adolescence which may predict later overweight, and information on possible BMI-specific differences, may provide important new data for use in intervention programmes targeting young adolescents.

The aim of the present study was to investigate BMI-specific associations between health-related behaviours (eating patterns, physical activity and screen time) in early adolescence (7th grade, ages 12–13 years) and the likelihood of being overweight in mid-adolescence (10th grade, ages 15–16 years) in a sample of Norwegian adolescents.

## Methods

### Participants and study design

The present data were obtained from a longitudinal study of diet, physical activity and BMI development in schoolchildren in Telemark County, Norway. Data collection took place in the spring of 2010 and follow-up in the spring of 2013. The children were at the last stage of primary school – 7th grade (mean age 12·7 (sd 0·3) years) – at baseline and the last stage of lower secondary school –10th grade (mean age 15·5 (sd 0·3) years) – at the time of follow-up. The present analyses incorporate parent-reported data on children’s dietary habits, daily physical activity and screen time, as well as background variables recorded in the 7th grade and weight and height data noted in the 7th and 10th grades. The questionnaire used for parental reports was initially pre-tested on a sample of parents and followed up by means of qualitative interviews^(^ ^26^ ^)^. The weights and heights of the adolescents were measured by public health nurses at each school at both collection points.

The detailed methods for data collection in the 7th grade have been described previously^(^ ^18^ ^,^ ^27^ ^)^. In brief, all 104 primary schools in Telemark County were invited to participate and fifty-three agreed. In total, 1503 children and their parents were invited and 1095 (73 % of those invited) participated, representing about half of the county’s 7th grade pupils. A similar procedure was used in the 10th grade. All secondary schools in Telemark County (thirty-nine) were included, but only 20 % of the invited parents completed the study questionnaire in the 10th grade.

**Fig. 1:**
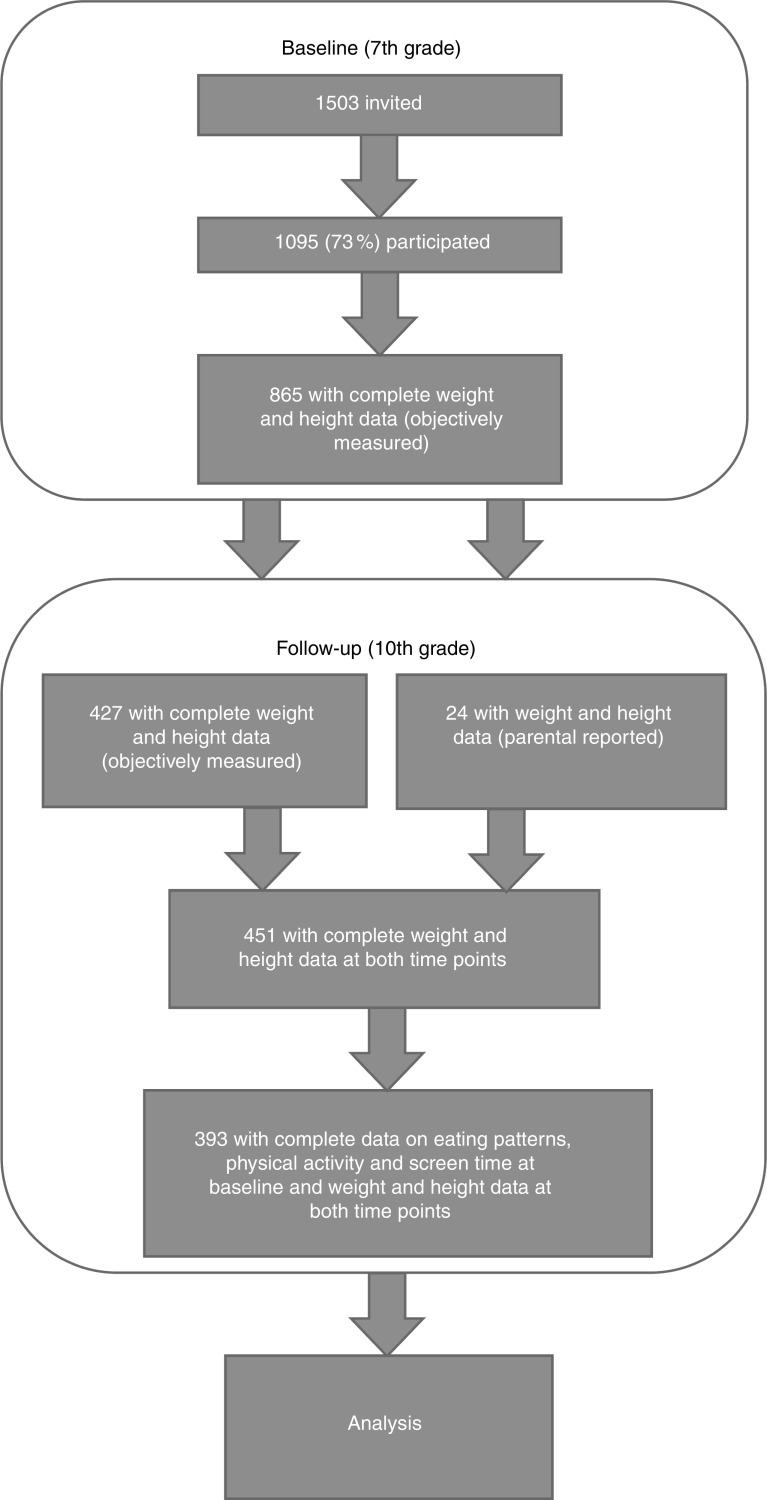
Overview of participants at baseline (in 7th grade, ages 12–13 years) and follow-up (in 10th grade, ages 15–16 years)

In total, weight and height measurement were conducted for 865 children in the 7th grade. Weight and height data were available for 427 (49 %) of these at follow-up (in the 10th grade). To expand the number of children with complete weight and height data, parental reports on weight and height in the 10th grade were added in cases where objectively measured data were missing (*n* 24), resulting in 451 individuals with complete weight and height data. Complete data for the present analyses (eating patterns, physical activity and screen time in the 7th grade, and weight and height data from both collection points) were available for 393 children (Fig. 1).

The study was conducted in accordance with the guidelines laid down in the Declaration of Helsinki and all procedures involving human subjects were the approved by the Regional Committee for Ethics in Medical Research and the Norwegian Data Inspectorate.

### Dietary information

The children’s food and drink intake and meal frequencies were reported by their parents using a retrospective FFQ, which asked about habitual daily consumption of forty food items, thirteen types of snacks (between meals), eleven types of drinks and five meals (breakfast, lunch, afternoon meal (light meal/snack between lunch and dinner), dinner and evening meal) in the previous 6 months. The questionnaire was based on a short validated FFQ developed for use with children in the 4th and 8th grades in Norway with help from parents^(^ ^28^ ^)^. The results indicated that the short FFQ was able to identify high and low food intakes and offered moderate scope for ranking individuals according to food intake. In the present study, the FFQ was modified to include several additional dietary questions and was pre-tested on a sample of parents and followed up by means of qualitative interviews^(^ ^26^ ^)^. The resulting extended FFQ is appropriate for exploring overall dietary habits by means of eating patterns. The additional questions covered meat and meat products, fish and fish products, different types of breads (white/wholemeal), breakfast cereals (with/without sugar), rice, pasta, spreads, milk products (full-fat/low-fat drinks, yoghurts and cheeses), a broad selection of commonly consumed soft drinks (with/without sugar) and different snack products (high-/low-energy)^(^ ^18^ ^)^. There were seven response options for foods and drink items: ‘rarely/never’, ‘1–3 times a month’, ‘1–3 times a week’, ‘4–6 times a week’, ‘once a day’, ‘twice a day’ and ‘3 or more times a day’. Meal frequencies were registered for the five main meals with eight response alternatives ranging from ‘never/rarely’ to ‘daily’. All dietary variables were used and included in the principal component analysis (PCA) as separate input variables^(^ ^18^ ^)^.

Four distinct eating patterns have previously been identified using PCA of total reported dietary responses in the 7th grade^(^ ^18^ ^)^. The eating patterns were named after the ingredients of each eating pattern, as follows: (i) ‘junk/convenient’, characterised by high-energy processed fast foods, refined grains, cakes and sweets; (ii) ‘varied Norwegian’, characterised by food items typical of a traditional Norwegian diet, including fruits and vegetables, brown bread, fish, water and regular breakfast and lunch, close to official nutritional guidelines; (iii) ‘snacking’, characterised by sugar-rich snack items and drinks, low intake of vegetables and brown bread, low frequency of breakfast and dinner and high frequency of eating between meals; and, finally, (iv) ‘dieting’, characterised by foods and drinks often associated with weight control, like artificially sweetened drinks and other ‘light’ products.

PCA is a well-established method for deriving dietary patterns in a population^(^ ^29^ ^,^ ^30^ ^)^. The method constructs new linear factors (patterns) by grouping together correlated dietary variables. As we used meals in addition to food consumption frequencies as input variables in the PCA, the factors were denoted as ‘eating patterns’ rather than ‘dietary patterns’. The coefficients defining the factors are called factor loadings and are the correlations of each dietary variable with the factors. Factor scores were created by multiplying factor loadings with the corresponding standardised value for each food/drink/meal and summing across all dietary variables^(^ ^18^ ^)^. For each individual, the factor scores indicate the extent to which the person’s diet conformed to the respective eating patterns. Positive factor scores indicate a high intake of foods, drinks and meals included in the respective pattern, while negative factor scores indicate a low intake^(^ ^29^ ^,^ ^30^ ^)^.

The factor scores for each pattern were divided into tertiles, then further into two categories: ‘low intake’ (lower tertile) and ‘moderate to high intake’ (middle and upper tertiles). The variances explained by the junk/convenient, varied Norwegian, snacking and dieting eating patterns were 8, 6, 4 and 3 %, respectively. All the identified eating patterns were applied unchanged as independent variables in the present analysis.

### Physical activity and screen time

The physical activity of the children was measured by asking parents, ‘For how long is your child physically active (sweaty or breathless) on a normal day?’ The possible answers were ‘one hour or more’ and ‘less than one hour’. The categories reflect the officially recommended amount of daily physical activity for children and adolescents (at least 1 h of MVPA daily).

Total screen time was calculated from parental reports of the number of hours their children typically spent on three different leisure-time screen-based activities (television and digital video disc/video viewing, playing video games and using a personal computer). The response options for each activity were: ‘not daily’, ‘<½ hour per day’, ‘½−1 hour per day’, ‘2–3 hours per day’, ‘4 hours per day’ and ‘>4 hours per day’. The response categories were given the values 0, 0·25, 0·75, 2·5, 4 and 5, respectively. The three screen-based activities were then amalgamated into a single overall screen-time variable (mean score for daily screen time). The Norwegian health authorities generally recommend a low level of sedentary activity, but do not prescribe specific time limits. A limit of 2 h of screen time daily is commonly used as an indicator of health risk^(^ ^31^ ^)^, but most children and adolescents in Western countries far exceed this limit^(^ ^9^ ^)^. To create sufficiently large categories and ensure the use of what we considered a realistic time limit for ordinary families, we chose a cut-off value of 3 h/d, close to the median (3·25). The defined categories were ‘≤3 h/d’ and ‘>3 h/d’ (representing two groups with approximately equal numbers of participants) and the variable was labelled ‘screen time’.

### Recommended diet and moderate-to-vigorous physical activity

To study the possibility of a favourable combined effect of a varied diet and recommended MVPA, a combined variable incorporating the varied Norwegian eating pattern and MVPA was constructed. The categories were ‘moderate to high intake of the varied Norwegian eating pattern and MVPA ≥1 h/d’ and ‘low intake of a varied Norwegian eating pattern and/or MVPA <1 h/d’. The variable was labelled ‘recommended diet and MVPA’.

### BMI categories

The weights and heights of the adolescents were measured by public health nurses at each school at both collection points. The adolescents were weighed wearing light clothing (i.e. trousers, T-shirt, socks), using calibrated, electronic scales measuring in 100 g increments. In addition, parent-reported weight and height were collected to include children with missing objective data in the 10th grade (twenty-four children; 6 % of the total sample). Adolescent BMI categories were calculated using the International Obesity Task Force cut-off points (underweight, normal weight, overweight, obese), based on growth curves and BMI of 17·0, 25·0 and 30·0 kg/m^2^ at age 18 years^(^ ^32^ ^,^ ^33^ ^)^. The respective cut-off points for boys and girls aged 12·5 and 15·5 years were used. Due to small numbers, we included underweight children in the normal-weight group and obese children in the overweight group.

### Adjustment variables

In addition to dietary reports and information on physical activity and screen time, parents reported the children’s gender, their own educational level, family income and own height and weight.

Paternal and maternal educational level was divided into three categories: ‘primary and lower secondary education’ (basic: 10 years or less), ‘upper secondary education’ (an additional 3–4 years) and ‘university or university college’.

Family income was divided into three categories: ‘both parents <NOK 300 000’, ‘one parent ≥NOK 300 000’ and ‘both parents ≥NOK 300 000’ (where NOK is Norwegian kroner and NOK 300 000=€40 849 as at 12 December 2012).

The parental BMI category was calculated based on self-reported height and weight and the International Obesity Task Force cut-off points for adults (overweight at BMI ≥25·0 kg/m^2^)^(^ ^34^ ^)^.

### Statistical analyses

Differences in eating patterns, physical activity, recommended behaviour and screen time between children of normal weight and overweight children in the 7th grade were analysed using cross-tabulation and Pearson’s *χ* ^2^ test (Fisher’s test).

We used multiple logistic regression analysis to: (i) associate individual health-related behaviours (individual eating patterns, MVPA and screen time) in the 7th grade (independent variables) with the likelihood of being overweight in the 10th grade (dependent variable); and (ii) associate recommended diet and MVPA and screen time (independent variables) with the likelihood of being overweight in the 10th grade (dependent variable). Both analyses were stratified by BMI category at baseline (7th grade). The independent variables were adjusted for each other in the respective models. Odds ratios with 95 % confidence intervals were calculated for the likelihood of being overweight in the 10th grade. We applied forward conditional selection to include available adjustment variables significantly associated with the respective dependent variables in the models. Available adjustment variables were child gender, maternal and paternal education, family income, and maternal and paternal BMI category.

Only participants for whom complete data on all health-related behaviours (eating patterns, physical activity and screen time in the 7th grade) and BMI were available from both collection points (*n* 393) were used in the analyses. Behavioural data in the 10th grade were not included, due to the small number of respondents. Respondents with missing adjustment variable values were not excluded from the analysis, but included with ‘missing’ as a separate adjustment variable category in relevant cases.

For all tests, *P*<0·05 was considered significant. The questionnaires were scanned by Eyes and Hands (Readsoft Forms, Helsingborg, Sweden) and all the statistical analyses were carried out using the statistical software package IBM SPSS Statistics for Windows, Version 21.0.

## Results

The proportions of children who were overweight were 14 % at baseline and 15 % at follow-up. In the 10th grade, twenty-three of 339 (7 %) children of normal weight had become overweight during the time period, while seventeen of fifty-four (31 %) overweight children had achieved a normal weight (Table 1). Of the 393 participants, 46 % were girls and 54 % were boys. Some 52 % of mothers and 40 % of fathers were registered in the highest educational category, ‘university or university college’. In total, 46 % of parents were registered in the highest category of family income. Further family and child characteristics of the 7th grade sample are specified in Table 2.

**Table 1:** Changes in BMI categories from 7th grade (ages 12–13 years) to 10th grade (ages 15–16 years) among Norwegian adolescents (*n* 393), Telemark County, spring 2010–spring 2013

		BMI category in 10th grade			
		Normal weight		Overweight	
		*n*	%	*n*	%
BMI category in 7th grade	Normal weight (*n* 399; 100 %)	316	93	23	7
	Overweight (*n* 54; 100 %)	37	69	17	31

**Table 2:** Family and child characteristics in 7th grade (ages 12–13 years) among Norwegian adolescents (*n* 393), Telemark County, spring 2010

Characteristic	Category	*n*	%
Maternal education	Primary and lower secondary	32	8
	Upper secondary	138	35
	University or university college	204	52
	Missing	14	5
Paternal education	Primary and lower secondary	42	11
	Upper secondary	167	42
	University or university college	156	40
	Missing	28	7
Maternal BMI category	Normal weight	236	60
	Overweight	122	31
	Missing	35	9
Paternal BMI category	Normal weight	131	83
	Overweight	210	53
	Missing	52	13
Family income	Both parents <NOK 300 000	28	7
	One parent ≥NOK 300 000	176	45
	Both parents ≥NOK 300 000	182	46
	Missing	7	2
Child gender	Boy	181	46
	Girl	212	54

NOK, Norwegian kroner (NOK 300 000=€40 849 as at 12 December 2012).

At baseline (in the 7th grade), a significantly higher proportion of normal-weight adolescents than overweight adolescents had a moderate to high intake of a varied Norwegian eating pattern (74 % *v*. 57 %) and MVPA ≥1 h/d (43 % *v*. 26 %; Table 3). No significant differences were observed between BMI category groups with regard to the combination of recommended diet and MVPA, other eating patterns or screen time (Table 3).

**Table 3:** Health-related behaviours (eating pattern, physical activity and screen time) of normal-weight and overweight Norwegian adolescents (*n* 393) in the 7th grade (ages 12–13 years), Telemark County, spring 2010

		Normal weight (*n* 339)		Overweight (*n* 54)		
Behaviour	Category	*n*	%	*n*	%	*P*
Junk/convenient eating pattern	Low intake	105	31	17	31	1·000
	Moderate to high intake	234	69	37	69	
Varied Norwegian eating pattern	Low intake	88	26	25	46	**0**·**003**
	Moderate to high intake	251	74	29	54	
Snacking eating pattern	Low intake	113	33	22	41	0·285
	Moderate to high intake	226	67	32	59	
Dieting eating pattern	Low intake	121	36	12	22	0·063
	Moderate to high intake	218	64	42	78	
MVPA	<1 h/d	194	57	40	74	**0**·**024**
	≥1 h/d	145	43	14	26	
Screen time	≤3 h/d	160	48	27	50	0·884
	>3 h/d	179	52	27	50	
Recommended diet and MVPA	Low intake of varied Norwegian eating pattern and/or MVPA <1 h/d	225	66	42	78	0·116
	Moderate to high intake of varied Norwegian eating pattern and MVPA ≥1 h/d	114	34	12	22	

MVPA, moderate-to-vigorous physical activity. Significant *P* values are shown in bold.

Screen time of >3 h/d in the 7th grade was positively associated with being overweight in the 10th grade, but only among adolescents who were initially of normal weight (adjusted OR=2·7; 95 % CI 1·0, 7·3; Model 1, Table 4). No significant associations were observed between individual eating patterns or MVPA level in the 7th grade and subsequent overweight in either BMI category group, although several trends were apparent (Model 1, Table 4).

**Table 4:** Multiple logistic regression results (odds ratios and 95 % confidence intervals) for the associations between health-related behaviours (eating pattern, physical activity and screen time) in the 7th grade (ages 12–13 years) and the likelihood of being overweight in the 10th grade (ages 15–16 years), stratified by BMI category in the 7th grade, Norwegian adolescents (*n* 393), Telemark County, spring 2010–spring 2013

Health-related behaviour in		Normal weight (*n* 339)		Normal weight (*n* 339)		Overweight (*n* 54)		Overweight (*n* 54)	
the 7th grade	Category	OR_crude_	95 % CI	OR_adj_ *	95 % CI	OR_crude_	95 % CI	OR_adj_ *	95 % CI
Model 1									
Junk/convenient eating pattern	Low intake	1·0	Ref.	1·0	Ref.	1·0	Ref.	1·0	Ref.
	Moderate to high intake	1·0	0·4, 2·6	0·9	0·4, 2·3	2·8	0·8, 9·3	2·2	0·6, 8·6
Varied Norwegian eating	Low intake	1·0	Ref.	1·0	Ref.	1·0	Ref.	1·0	Ref.
pattern	Moderate to high intake	0·9	0·5, 1·4	0·8	0·3, 1·9	0·7	0·3, 1·4	0·5	0·1, 2·0
Snacking eating pattern	Low intake	1·0	Ref.	1·0	Ref.	1·0	Ref.	1·0	Ref.
	Moderate to high intake	0·9	0·4, 2·3	0·8	0·3, 2·0	2·1	0·6, 6·7	1·4	0·4, 5·1
Dieting eating pattern	Low intake	1·0	Ref.	1·0	Ref.	1·0	Ref.	1·0	Ref.
	Moderate to high intake	1·0	0·4, 2·5	1·0	0·4, 2·5	0·7	0·2, 2·9	1·0	0·2, 5·1
MVPA	<1 h/d	1·0	Ref.	1·0	Ref.	1·0	Ref.	1·0	Ref.
	≥1 h/d	0·7	0·3, 1·7	0·8	0·3, 2·0	0·3	0·1, 1·2	0·6	0·1, 2·7
Screen time	≤3 h/d	1·0	Ref.	1·0	Ref.	1·0	Ref.	1·0	Ref.
	>3 h/d	**2**·**8**	**1·1**, **7·3**	**2**·**7**	**1·0**, **7·3**	1·7	0·5, 5·4	1·1	0·3, 4·0
Model 2									
Recommended diet and MVPA	Low intake of varied Norwegian eating pattern and/or MVPA <1 h/d	1·0	Ref.	1·0	Ref.	1·0	Ref.	1·0	Ref.
	Moderate to high intake of varied Norwegian eating pattern and MVPA ≥1 h/d	0·9	0·3, 2·1	1·0	0·4, 2·5	**0**·**2**	**0·06**, **0·9**	**0**·**2**	**0·1**, **1·0**
Screen time	≤3 h/d	1·0	Ref.	1·0	Ref.	1·0	Ref.	1·0	Ref.
	>3 h/d	**2**·**8**	**1**·**1**, **7**·**3**	**2**·**8**	**1**·**1**, **7**·**3**	1·7	0·5, 5·4	1·2	0·3, 4·2

MVPA, moderate-to-vigorous physical activity; ref., reference category. Significant associations are shown in bold.

* OR_adj_ adjusted for all other health-related behaviours (eating pattern, physical activity and screen time). Available background variables were not significantly associated in the models.

Recommended diet and MVPA (moderate to high intake of a varied Norwegian eating pattern combined with MVPA ≥1 h/d) in the 7th grade was associated with a reduced likelihood of being overweight in the 10th grade (adjusted OR=0·2; 95 % CI 0·1, 1·0), but only among already overweight adolescents (Model 2, Table 4). As in Model 1, screen time of >3 h/d in the 7th grade was the only behaviour associated with an increased likelihood of subsequent overweight among adolescents who were initially of normal weight (adjusted OR=2·8; 95 % CI 1·1, 7·3; Model 2, Table 4).

None of the available background variables were significantly associated in the regression models. Running the same models with adjustments for all background variables did not significantly alter the estimates.

## Discussion

The main finding of the present study was that the association between health-related behaviours in early adolescence and the likelihood of being overweight in mid-adolescence was modified by baseline BMI category. A combination of a moderate to high intake of a varied Norwegian diet and recommended MVPA was associated with a reduced likelihood of subsequent overweight among initially overweight adolescents, while high screen time was associated with an increased likelihood of subsequent overweight among those who were initially of normal weight.

A direct comparison with other studies is difficult, due to different data collection methods and study designs, different age groups and varying measures of diet, physical activity and overweight/obesity. However, some similarities and differences can be noted.

Significantly more normal-weight than overweight adolescents had a moderate to high intake of a varied Norwegian eating pattern at baseline. Previous cross-sectional studies have reported better meal regularity, especially regular breakfast, among normal-weight children than children who are overweight^(^ ^35^ ^,^ ^36^ ^)^. However, demonstrating healthier food choices among children of normal weight than overweight children has proved difficult. Often, the opposite is evident, probably due to parental food restraints or adoption of healthy food choices to lose weight^(^ ^3^ ^,^ ^37^ ^)^. No significant indications of food restrictions were apparent in the overweight group, although the intake of dieting products was slightly higher than that observed in the normal-weight group.

Our results are in line with the majority of previous adolescent studies in which low physical activity has been linked with overweight^(^ ^4^ ^)^. The causes of lower physical activity among overweight youth may be compound, as low physical activity may act as both an antecedent and a consequence of BMI status^(^ ^4^ ^)^. Several studies indicate that overweight children may be reluctant to engage in physical activity due to a fear of teasing and peer victimisation^(^ ^4^ ^)^. This in turn may lead to social isolation and inactivity^(^ ^4^ ^)^. Although several cross-sectional studies^(^ ^4^ ^,^ ^6^ ^,^ ^24^ ^)^ have previously linked excessive screen time, especially television viewing, with overweight, null findings are also common^(^ ^4^ ^,^ ^6^ ^)^. In the present study, we observed equal screen time among normal-weight and overweight 7th graders.

Previous studies have reported BMI-specific differences in the association between individual health-related behaviours (recommended overall diet, physical activity and screen time) and overweight development in adolescents^(^ ^7^ ^,^ ^18^ ^,^ ^21^ ^–^ ^25^ ^,^ ^38^ ^)^. We have previously observed that already overweight children who adhered to a varied Norwegian eating pattern (a pattern close to the recommended) between the 4th and 7th grades were more likely to achieve normal weight in the 7th grade than overweight children with declining adherence to this pattern^(^ ^18^ ^)^. No comparable favourable effect on overweight development was seen among children of normal weight. Comparably, a recently published Australian study of 9–12-year-old children concluded that improvement in diet quality in line with official advice was associated with improvement in BMI *Z*-scores after 3 years, but only among already overweight children^(^ ^22^ ^)^. It has also been reported that adolescents with normal weight may be less vulnerable to unfavourable weight development due to the consumption of energy-rich foods than already overweight individuals, partly because overweight adolescents are less likely to compensate for the energy in fast food^(^ ^25^ ^)^. Moreover, previous studies have indicated differences in the associations between physical activity and adiposity in normal-weight as compared with overweight children^(^ ^21^ ^,^ ^38^ ^)^. High physical activity has been associated with a stable BMI and a decrease in body fat percentage in children of normal weight^(^ ^21^ ^)^ and an increase in BMI, body fatness and fat-free body mass in overweight children^(^ ^21^ ^,^ ^38^ ^)^. In the present study, high screen time appeared to be the only significant predictor of subsequent overweight, although – contrary to expectations based on previous studies^(^ ^6^ ^,^ ^7^ ^,^ ^23^ ^,^ ^24^ ^)^ – only among adolescents who were initially of normal weight.

In both our models, normal-weight 7th graders who spent more than 3 h on screen-time activities daily were almost three times more likely to be overweight in mid-adolescence than those who spent less than 3 h/d on such activities. The findings suggest that high screen time in early adolescence may be an important indicator of later overweight among normal-weight children, making normal-weight children with high screen times a particularly relevant group for targeted preventive measures. It seems likely that high screen time in early adolescence may be a risk factor in terms of subsequent unhealthy habits and weight gain, as screen time tends to track through adolescence^(^ ^13^ ^)^ and is often associated with unhealthy dietary patterns^(^ ^39^ ^)^. In our sample, high screen time was associated with a high intake of the snacking eating pattern at baseline (*P*<0·05, data not shown). However, adjustment for eating patterns and other baseline behaviours did not significantly influence the likelihood of subsequent overweight among initially normal-weight adolescents with high screen times, indicating an important independent relationship.

A combination of a moderate to high intake of a varied eating pattern and a recommended level of MVPA in early adolescence was associated with a markedly reduced likelihood of later overweight among already overweight adolescents. Overweight adolescents with this combination at baseline were five times less likely to remain overweight in mid-adolescence than other overweight adolescents, independently of other eating patterns and screen time. However, following a recommended diet and engaging in recommended physical activity in early adolescence did not predict favourable weight development among normal-weight individuals. The findings are in line with previous studies in which healthy dietary patterns have been associated with favourable BMI change among overweight individuals, but not persons who are initially of normal weight^(^ ^18^ ^,^ ^22^ ^,^ ^25^ ^,^ ^40^ ^)^. Nevertheless, the results indicate that establishing behaviour in line with current guidelines on diet and MVPA among overweight young adolescents may contribute to lasting adoption of a more favourable lifestyle and subsequent weight reduction.

The mechanisms behind the observed BMI-dependent differences may be compound. Previous studies have explained similar differences by gene–environment interactions, as different physiological responses to diet and physical activity/sedentary activity among overweight and normal-weight individuals may influence susceptibility to weight change^(^ ^6^ ^,^ ^41^ ^–^ ^43^ ^)^. Another explanation is differences in behaviour stability between overweight and normal-weight adolescents over the time studied. Our limited available responses on behavioural variables in the 10th grade (*n* 148) indicate moderate tracking of single food items (fruits, vegetables and wholegrain products), MVPA and screen time from the 7th to the 10th grade (*P*<0·05). This suggests some degree of behavioural tracking through the study period, but also indicates that a fair number of adolescents changed their behaviour between early and mid-adolescence. Due to the small numbers in the overweight group and low response rate at follow-up, we were unable to differentiate between BMI category groups to study possible differences further. To our knowledge, no studies exploring tracking of health-related behaviours in normal-weight *v*. overweight subjects are currently available. It can be speculated, however, that overweight adolescents, often under parental regulation, are more likely than their normal-weight peers to maintain a healthy diet and physical activity, and reduce sedentary activities, in order to lose weight. On the other hand, normal-weight adolescents, and their parents, may have a less concerned attitude towards weight gain and therefore pay less attention to controlling and modifying weight-related risk behaviours.

In line with current national recommendations on diet and physical activity, our results indicate that measures targeting overweight young adolescents and their parents should focus on a varied diet and recommended level of physical activity to reduce the likelihood of later overweight. Further, initiatives to reduce screen time are especially important to prevent subsequent overweight among normal-weight adolescents who spend much time on screen-based activities.

### Strengths and limitations

The present study has strengths, but also limitations that should be recognised. An important strength is the use of PCA-derived eating patterns as a measure of the children’s overall dietary and meal habits^(^ ^29^ ^)^. The FFQ included a wide range of commonly consumed food items, snack products, drinks and meals, resulting in robust factors (eating patterns) covering multiple items. The study also incorporated data on MVPA and screen time, allowing for investigation of independent individual behavioural variables considered relevant to overweight development in adolescence. Furthermore, the study included several background variables considered to be possible correlates with regard to health-related behaviour and overweight variability^(^ ^1^ ^,^ ^4^ ^,^ ^44^ ^–^ ^46^ ^)^, an important means of reducing potential bias due to residual confounding.

Another strength is the objective measurement of height and weight at both collection points. A small proportion of the weight and height data in the 10th grade was parent-reported. This may have led to some bias due to under-reporting in the 10th grade and this in turn may have influenced the prospective findings for these children. However, the parent-reported data related to only 6 % of the children (*n* 24) in the 10th grade and we therefore consider this problem to be limited. Separate analysis of participants for whom only objectively measured data were available did not result in any significant changes to the estimates.

A further strength is the study’s longitudinal perspective. However, determination of longitudinal changes in health-related behaviour and simultaneous change in overweight was impossible due to a low response rate and uncompleted questionnaires in the 10th grade. Unidentified changes in behaviour may therefore have influenced the observed associations. Future studies should ensure simultaneous collection of behavioural information and BMI data at all collection points.

It can be assumed that sociodemographic factors may influence an association between health-related behavioural variables and overweight. However, none of the available background variables (gender, parental education, family income and parental BMI) were significantly associated in the forward conditional selection models used, indicating independent relationships between the main variables. Using an alternative approach that incorporated all the adjustment variables did not change the estimates substantially (data not shown). Nevertheless, we cannot exclude the possibility that other sociocultural and environmental factors not taken into account here may have attenuated the examined associations. Physical home environment factors, like access to media devices, home surroundings and access to equipment for physical activity, may influence children’s physical activity and sedentary behaviour^(^ ^47^ ^)^. Social environment factors, particularly the role of parents, have previously been associated with children’s diet, physical activity and sedentary behaviour. Parental motives for food choices^(^ ^48^ ^)^, parental screen time^(^ ^49^ ^,^ ^50^ ^)^ and parental perceptions of children’s screen time and physical activity^(^ ^50^ ^)^ are examples of important factors not considered here.

Due to small numbers in the overweight group, we were unable to stratify by gender or social background in the regression analyses. Where the number of participants is limited, and statistical power is consequently weaker, only relationships with a strong impact will be detectable. This may explain why some expected relationships did not achieve significance and were observed only as trends in odds ratios.

We cannot exclude the possibility that parental dietary reports may have influenced the associations, for example by deliberate misrepresentations such as under-reporting of unhealthy items and/or over-reporting of healthy products^(^ ^51^ ^)^. However, the FFQ data were used to derive patterns reflecting dietary behaviour, which are less likely to be distorted by misreporting than estimated intakes of energy, nutrients and food amounts^(^ ^52^ ^)^. Nevertheless, bias due to errors of memory and deficient parental insight into eating outside the home cannot be excluded^(^ ^53^ ^)^. It is likely that the dietary data reflect the parents’ ‘dietary image’ rather than the true habitual diet of the adolescents^(^ ^51^ ^)^ and must therefore be treated as proxy reports. We have previously collected parent-reported dietary data on the same children at age 9–10 years^(^ ^27^ ^)^. The present study was a follow-up and parental reporting was chosen to avoid differing data collection conditions at the two collection times. A further aim was to reduce under-reporting, which is thought to be common among adolescents^(^ ^51^ ^)^. The reproducibility and validity of PCA-derived dietary patterns assessed using FFQ have previously been found to be comparable to those of patterns obtained using weighed dietary records^(^ ^54^ ^–^ ^56^ ^)^.

As for dietary information, parental proxy reports of physical activity and screen time may be prone to bias caused by misreporting due to incomplete insight into children’s activity levels, errors of memory, and deliberate misrepresentations and social acceptability^(^ ^57^ ^,^ ^58^ ^)^. We cannot exclude the possibility that parental misreporting in the 7th grade may have attenuated the observed effect. However, parental reports have been suggested to provide a more accurate assessment than children’s self-reporting of activity levels up to 12 years of age^(^ ^58^ ^)^ (the children in our study were 12·5 years old at baseline). Furthermore, due to the prospective design of the study, any parental misreporting at baseline is likely to be independent of changes in BMI status during the study period, making such bias of lesser concern.

Another possible limitation on the results is bias due to non-responders. However, no systematic participation bias was observed with regard to school size and location. The participating parents had a somewhat higher educational level and total family income than the Norwegian population in general. Further, we only analysed participants with complete data on behavioural variables in the 7th grade and BMI at both collection points. Because those with missing data on these variables did not differ substantially from the remaining informants with regard to background variables, we consider this problem to be limited. Data collection was restricted to one Norwegian county and therefore the results are not necessarily representative of the national population.

## Conclusion

The present study suggests BMI-specific associations between health-related behaviours in early adolescence and the likelihood of overweight in mid-adolescence. The results indicate that establishing a varied diet in combination with a recommended level of physical activity in early adolescence may support favourable weight development among already overweight adolescents, while reducing screen time may be the most important measure to prevent subsequent overweight in adolescents who are initially of normal weight. The findings suggest that BMI-specific interventions are important to reduce overweight among adolescents.

To study tracking of health-related behaviours related to BMI-specific weight development, future longitudinal studies should incorporate simultaneously collected behavioural information and BMI at baseline and follow-up. To investigate the observed associations further, it will be desirable to repeat the analysis on a larger study sample to confirm our results and to allow investigation of possible gender differences and social inequalities.
